# The Role of Irisin in Exercise-Mediated Bone Health

**DOI:** 10.3389/fcell.2021.668759

**Published:** 2021-05-04

**Authors:** Lifei Liu, Jianmin Guo, Xi Chen, Xiaoyang Tong, Jiake Xu, Jun Zou

**Affiliations:** ^1^School of Kinesiology, Shanghai University of Sport, Shanghai, China; ^2^Department of Rehabilitation, The People’s Hospital of Liaoning Province, Shenyang, China; ^3^School of Sports Science, Wenzhou Medical University, Wenzhou, China; ^4^School of Biomedical Sciences, University of Western Australia, Perth, WA, Australia

**Keywords:** irisin, exercise, bone health, skeleton, mechanical force

## Abstract

Exercise training promotes physical and bone health, and is the first choice of non-drug strategies that help to improve the prognosis and complications of many chronic diseases. Irisin is a newly discovered peptide hormone that modulates energy metabolism and skeletal muscle mass. Here, we discuss the role of irisin in bone metabolism via exercise-induced mechanical forces regulation. In addition, the role of irisin in pathological bone loss and other chronic diseases is also reviewed. Notably, irisin appears to be a key determinant of bone mineral status and thus may serve as a novel biomarker for bone metabolism. Interestingly, the secretion of irisin appears to be mediated by different forms of exercise and pathological conditions such as diabetes, obesity, and inflammation. Understanding the mechanism by which irisin is regulated and how it regulates skeletal metabolism via osteoclast and osteoblast activities will be an important step toward applying new knowledge of irisin to the treatment and prevention of bone diseases such as osteolysis and other chronic disorders.

## Introduction

Exercise training is well known to have beneficial effects on physical fitness and bone health. Physical activity reduces the risk of many chronic diseases and aging-related disorders, such as diabetes mellitus, hypertension, obesity, and osteoporosis (Farmer, 2019). Lack of physical activity, such as resembling bedridden patients, causes a lack of mechanical stimulation, leading to an imbalance of bone formation and resorption and a speedy loss of bone mass (Benedetti et al., 2018). Proper high impact physical activity enables bone to respond positively, and improve the renew of bone metabolism, bone mineral density (BMD) and structural properties in the loaded bone regions and whole body (Beck et al., 2017; Gomez-Bruton et al., 2017; Okubo et al., 2017). Meanwhile, regular physical activity ameliorates bone health status and reduces the risk of trauma fragility fracture and secondary functional disfunction (Kemmler et al., 2015; Troy et al., 2018).

Irisin is a newly discovered peptide hormone of 112 amino acids, which is the extracellular domain of a transmembrane protein fibronectin type III domain-containing 5 (FNDC5). FNDC5 and its cleaved circulating form irisin are positively correlated with an active lifestyle (Tenorio et al., 2017). Vigorous-intensity physical activity had a high serum level of irisin (Morelli et al., 2020), which positively related to bone mechanical properties (Zhang et al., 2020). Furthermore, treatment with irisin is found to improve BMD and biomechanical properties in murine models (Colaianni et al., 2015). Here we review irisin as a key factor linking exercise and bone health and discuss the different roles of irisin in musculoskeletal system and some chronic disease conditions mediating bone metabolism in the context of exercise.

## Role of Exercise in Regulating Bone Metabolism

Exercise regulates bone metabolism, mainly through direct action (mechanical force) and indirect action (nerve and hormone regulation). Mechanical force acts on bone via ground reaction and muscle contraction forces (Usui et al., 2003). Loss of mechanical force, such as hindlimb murine, the trabecular bone volume was reported significantly reduced, and subsequent reloading results in a significant increase in trabecular bone volume (Colaianni et al., 2017; Cunningham et al., 2018). The central nervous system, especially the cerebral cortex and cerebellum regulates the neuromuscular and musculoskeletal systems, leading to the activation and precise adjust of bones (Cardozo and Graham, 2017). While, the hypothalamus influences bone is mainly by regulating the secretion of pituitary hormones (Dimitri and Rosen, 2017). Additionally, the autonomic nervous system, including the sympathetic (negative regulator) and parasympathetic (positive regulator) nervous systems has also been found to affect bone metabolism (Houweling et al., 2015; Idelevich and Baron, 2018).

Irisin is found to regulate bone cell metabolism by mechanical force (Storlino et al., 2019), and to increases both cortical and trabecular BMD subsequently (Colaianni et al., 2017). Additionally, muscle strength is found to be enhanced after irisin treatment (Reza et al., 2017b). Moreover, irisin participates in the regulation process of the hypothalamus and the autonomic nervous system (Scalzo et al., 2014; Poretsky et al., 2017) and interacts with hormones related to bone metabolism (Kim and Kim, 2018).

### Regulation of Bone Cell Metabolism

Bone physiological functions are mainly maintained by the activity of bone cells such as osteoblasts, osteoclasts and osteocytes (Clarke, 2008). Osteoblast-led bone formation and osteoclast-led bone resorption maintain bone homeostasis simultaneously (Kular et al., 2012). Osteocytes are the chief mechanosensory cells, and stellate cells embedded in calcified bone matrix, which accounts for over 90% of the bone cells (Klein-Nulend et al., 1995, 2012; Bonewald, 2011).

During high impact and rapid loading physical exercise (Beck et al., 2017), ground reaction forces exert mechanical forces on the bones (Usui et al., 2003), and the deformation of the bone matrix drives an interstitial fluid flow, which surrounds the osteocytes (Knothe Tate et al., 2000). Stimulated by the fluid mechanical signals, osteocytes regulate a cascade of biochemical responses (Kulkarni et al., 2012). Simultaneously, osteocytes convert mechanical strain into regulatory signals to stimulate the adaptive response of osteoclasts, thereby activating the bone resorption and subsequent formation process (Bonewald, 2011; Troy et al., 2018).

Irisin prevents the decrease of living osteocytes and the increase of empty cavities due to disuse (Storlino et al., 2019). Previous studies have shown that mechanical load leads to an increase of Wnt protein in osteocytes, thereby activating the classic Wnt signaling pathway, adjusting its sensitivity to mechanical load in the feedback loop (Tu et al., 2012). Corresponding, FNDC5 knockdown cells show downregulated Wnt expression (Ma et al., 2019). One study reveals that irisin increases survival of osteocytes by activating the mitogen-activated protein kinase (MAPK) extracellular signal-regulated kinase 1 (ERK1) and ERK2, which increases the expression of the transcription factor activating transcription factor 4 (ATF4) through an ERK-dependent pathway. Besides, irisin has been found to inhibit apoptosis in osteocytes (Storlino et al., 2019).

The osteoblast is the target of irisin as well. Irisin enhances osteoblast differentiation, proliferation, mineralization, and upregulates the expression of transcription regulators, such as runt-related transcription factor-2 (Runx2) and osterix (Colaianni et al., 2015; Zhang et al., 2017). Recent studies reveal that the osteogenic effect is mediated by irisin through activation of the p38 MAPK and ERK (Qiao et al., 2016), and differentiation promotion effect might be associated with activation of AMPK AMP-activated protein kinase (AMPK)-α signaling (Ye et al., 2020) and Wnt/β-catenin pathway (Robinson et al., 2006). Other studies indicate that the proliferation of osteoblasts can be promoted by irisin via enhancing aerobic glycolysis (Zhang et al., 2018), and the osteoblast apoptosis is suppressed by irisin via upregulating nuclear factor E2-related factor 2 (Nrf2), inhibiting pyrin domain containing protein 3 (NLRP3) inflammasome and lowering the content of inflammatory factors, which cause the reduction of the incidence of postmenopausal osteoporosis (Xu et al., 2020). Besides, recent studies show that irisin not only stimulates autophagy but also downregulates a senescence effector p21 to promote osteoblastogenesis and maintain the activity of osteoblast (Chen et al., 2020; Colaianni et al., 2021).

Osteoclast formation and differentiation are significantly reduced by irisin treatment (Zhang et al., 2017), and nuclear factor κB (NF-κB) ligand (RANKL) was found to be a key factor (Kawao et al., 2018). Irisin suppresses the receptor activator of RANKL/nuclear factor of activated T cells (NFAT) c1 pathway, thereby inhibits osteoclast formation in mouse bone marrow cells (Zhang et al., 2017; Kawao et al., 2018). A study of FNDC5 knockout mice indicates that the higher expression of RANKL and increased number of osteoclasts cause a decrease in bone strength and bone mass (Luo et al., 2020).

### Potential Role in Regulating the Crosstalk Between Muscle and Bone

Irisin was originally found to be secreted by muscle cells (Bostrom et al., 2012), and muscle contraction increases irisin secreted during exercise (Archundia-Herrera et al., 2017). Muscle is the major tissue of irisin expression, although it is also expressed in small amounts in bone, brain and other tissues (Colaianni et al., 2015). Muscle force itself is capable of providing sufficient stimulation to make bones respond (Judex and Rubin, 2010). Some investigations show that bone density is significantly related to muscle strength (Nordstrom et al., 1998). For example, the jumping force is positively correlated with increased bone mineral content (BMC) in the tibial cortex (Zengin et al., 2017), while, the decline of muscle function, such as sarcopenia, can lead to bone mass loss (Bonewald, 2019). Besides, the optimization of muscle strength, balance, and mobility brought by exercise can minimize the risk of falls and subsequent fractures, which is especially important for people at high risk of falls (Beck et al., 2017).

Irisin is positively correlated with muscle mass (Kim et al., 2016) and muscle strength, such as hand grip strength and leg strength (Martinez Munoz et al., 2019). Studies have established that the injection of irisin in murine induces significant hypertrophy of skeletal muscle and enhances muscle strength (Reza et al., 2017b), even reduce necrosis and fibrotic tissue (Reza et al., 2017a). The effect of irisin on hypertrophy is due to muscle stem cell activation and enhanced protein synthesis (Reza et al., 2017b). Muscle movement also induces peroxisome proliferator-activated receptor-γ coactivator 1 α (PGC1α), a transcriptional coactivator (Handschin and Spiegelman, 2008). PGC1α in muscle is reported to stimulate an increase in FNDC5 expression *in vitro* and *in vivo* (Bostrom et al., 2012). A recent study indicates that exercise increases mitochondrial fission and selective autophagy by PGC1a/FNDC5/irisin pathway, and promotes recovery of ischemic muscle (He et al., 2020). These studies suggest that irisin may play an important role in exercise relieving skeletal muscle atrophy, thereby maintaining bone load and bone mass.

### Exercise-Mediated Cell Factors in Bone Metabolism

Some cell factors are regulated by irisin during exercise to balance bone metabolism. Bone cells, especially osteocytes modify the production of a large number of signaling molecules when triggered by mechanical stimulation (Janik et al., 2018). At the same time, some central nervous system related hormones are also changed due to exercise, affecting bone metabolism (Kim and Kim, 2018; Liu et al., 2021).

Sclerostin is a protein produced by osteocytes, and causes endogenous inhibition of bone formation, which regulates bone remodeling (van Bezooijen et al., 2004). People who have more physical activities are tested as less sclerostin, and regular physical training results in a significant decrease of sclerostin level (Cheung and Giangregorio, 2012; Janik et al., 2018). Sclerostin is found to be a Wnt antagonist and blocks the Wnt/β-catenin signaling pathway (Singh et al., 2019). Therefore, sclerostin inhibitors can be expected to increase osteoblastogenesis (Lewiecki, 2011). Researches indicate that circulating irisin and sclerostin are highly negatively correlated (Colaianni et al., 2017). Additionally, irisin treatment inhibits the increase of sclerostin and restores osteoblastogenesis (Colaianni et al., 2017). On the contrary, one study reveals that irisin treatment upregulates sclerostin expression in osteoin-like cells (MLO-Y4) in a dose-dependent manner, and knockout of FNDC5 prevents ovariectomy mice from BMD loss by inhibiting osteolysis and bone resorption. The conflicting conclusion may be related to the different regulatory effects of irisin on osteogenesis and osteoclastogenesis under different conditions (Kim et al., 2018). This situation may be similar to the bidirectional regulation of bone by parathyroid hormone, in which intermittent administration leads to bone formation, while continuous administration causes bone loss (Rattanakul et al., 2003).

Leptin has been reported to regulate bone metabolism mainly by acting on the brain, especially via the hypothalamus and sympathetic nervous system (Motyl and Rosen, 2012; Reid et al., 2018). Leptin is a negative regulator of bone, and multiple lines of evidence show that high bone mass phenotype can be caused by leptin gene deletion accompanied with a massive increase in bone formation (Ducy et al., 2000; Karsenty, 2006). In addition, intraventricular injection of leptin reduces bone mass and volume by increasing osteoclast activity (Ducy et al., 2000). Exercise decreases leptin level, evidence suggests that running wheels exercise reduces circulating leptin levels in both adults and adolescents rats (Soch et al., 2016). Notably, circulating irisin and leptin are positively related in children and adults (Palacios-Gonzalez et al., 2015; Pena-Bello et al., 2016). In a study of rats, intraperitoneal irisin injections decreased the leptin level in circulation (Tekin et al., 2017). Interestingly, leptin has also been found to up-regulate the expression of FNDC5 through a nitric oxide-dependent mechanism (Rodriguez et al., 2015).

Brain-derived neurotrophic factor (BDNF) is found to be more expressed in bone than in the brain, as a neuroprotective factor (Camerino et al., 2016; Kowianski et al., 2018), and is involved in regulating the formation and fracture healing process of bone (Kilian et al., 2014). A report shows that BDNF promotes bone marrow mesenchymal stem cells osteogenesis by binding to the tropomyosin-related kinase B (TrkB) receptor, downstream Erk1/2 phosphorylation, and BDNF indirectly promotes osteogenesis by increasing neurogenesis as well (Liu et al., 2018). Exercises may have a regulatory effect on BDNF secretion, as studies reveal that a 3-months crossfit training increases BDNF level in young people (Murawska-Cialowicz et al., 2015), while sedentary rats have lower BDNF than the exercised ones, both young and aged (Belviranli and Okudan, 2018). Analogously, overexpression of irisin significantly upregulates BDNF expression, while irisin interference significantly downregulated the level of BDNF (Huang et al., 2019). To date, there is a lot of uncertainty in the role of irisin in cell factors expression level, which requires further research.

## Role of Exercise in Regulating Chronic Diseases

Some chronic diseases, such as diabetes mellitus (Mahapatra et al., 2016), inflammatory bowel diseases (Ali et al., 2009), hyperthyroidism (Novack, 2003), and relative adiposity (Dolan et al., 2017) are shown to be associated with low BMD and bone loss (Amin et al., 2011; Dimitri and Rosen, 2017). Exercise is the first line for treating various diseases, which also improves the prognosis and complications (Jin et al., 1999). The previous studies have established that exercise and irisin promote osteogenesis in some chronic diseases (Dieli-Conwright et al., 2018; Palermo et al., 2019).

### Diabetes Mellitus

Type 1 diabetes mellitus (T1DM) has an important link with osteoporosis which begins in childhood, and leads to lower peak bone mass and high risk of osteoporotic fractures in adults (Weber et al., 2015; Devaraja et al., 2020). Poor glycemic control and glycated hemoglobin (HbA1c) are found to be negatively correlated with BMD (Fuusager et al., 2019). Studies of diabetic mellitus rats demonstrate that running exercise increases the irisin level, glycemic control, bone mass and muscle strength, probable due to the activation of the Wnt/β-catenin signaling pathway and decreased systemic inflammatory process (Andrade et al., 2018; Yang et al., 2018; Sadeghipour et al., 2020).

Irisin levels are indicated significantly correlated negatively with HbA1c, years of diabetes, and positively associated with better glycemic control and bone health in TD1M children (Kurdiova et al., 2014; Faienza et al., 2018; Gouda et al., 2018). Furthermore, persistent subcutaneous perfusion of irisin improves insulin sensitivity, reduces fasting glycemia by inhibiting gluconeogenesis via phosphoinositide 3-kinase (PI3K)/serine/threonine kinase (Akt)/forkhead box transcription factor O1 (FOXO1) mediated phosphoenolpyruvate carboxykinase (PEPCK) (Liu et al., 2015). A recent study shows that irisin regulates glucose metabolism by promoting hepatic glycolysis and inhibiting hepatic gluconeogenesis (Yang et al., 2020). Therefore, irisin may improve bone metabolism of diabetic patients by regulating glycemic levels through exercise.

### Obesity

Some studies indicate that overweight children have lower bone mass than normal weight children, relative to their size and poorer bone structure parameters (Goulding et al., 2000; Farr et al., 2010). Another report shows bone strength is related to lean mass rather than fat mass (Bogl et al., 2011), and excess fat seems to limit the effect of lean mass on bone maturation (Farr and Dimitri, 2017).

Exercise can alleviate bone loss caused by obesity. A recent study suggests that subsequent 8 weeks of swimming relieves the reduced BMD, bone microstructure, and bone metabolic factors on obese rats (Kang et al., 2019). In obese breast cancer survivors, whole body and trochanter BMD have an upward trend after a 4-months exercise (Dieli-Conwright et al., 2018). Although the relevance to gender is unclear (Anastasilakis et al., 2014; Ruan et al., 2019), circulating irisin is shown positive correlated with adiposity indices, such as percent body fat and fat mass (Jang et al., 2017). Notably, a 6-months moderate physical exercise increases the irisin level, decreases body mass index and waist circumferences in obese men (Rashid et al., 2020). Therefore, irisin may be an important factor in maintaining the bone health of obese people.

### Inflammation

Lack of exercise also activates the inflammatory pathway network, which promotes the development of a cluster of diseases (Pedersen, 2009). Chronic inflammatory diseases cause excessive bone absorption and impaired bone formation, leading to periarticular and systemic bone loss (Straub et al., 2015; Metzger et al., 2019). Exercise promotes irisin expression and induces anti-inflammatory effects (Wiecek et al., 2018). Serum irisin levels are negatively correlated with inflammation-related symptoms, such as disease duration, severity evaluation, and stiffness duration in rheumatoid arthritis patients (Gamal et al., 2019). Anti-inflammatory property of irisin is associated with the downregulation of the Toll-like receptor 4 (TLR4)/myeloid differentiation primary response protein 88 (MyD88) downstream pathway and decreased the phosphorylation of NF-κB, consequently decreased phosphorylation and activation of crucial pro-inflammatory cytokines (Mazur-Bialy et al., 2017). Recently, in the study of a rat model of disuse osteoporosis, irisin treatment increases the bone formation rate of unloading hindlimbs and reduce the expression of pro-inflammatory factors such as tumor necrosis factor (TNF)-α and Interleukin (IL)-17 (Metzger et al., 2020).

## Potential Development of Irisin as Therapeutic Agent and Biomarkers for Bone Diseases

Increasing the level of physical activity is considered to be the preferred non-pharmacological intervention for the prevention and treatment of chronic bone diseases (Compston et al., 2017). Some resistance trainings during early life improve BMD and bone structural properties, and have a direct preventive effect on bone diseases in later life (Gomez-Bruton et al., 2017), and irisin also plays a key role in this process (Elizondo-Montemayor et al., 2018). Although different types of exercise training have conflicting results on irisin, most studies suggest that high-moderate intensity (Tsuchiya et al., 2014; Rashti et al., 2019; Torre-Saldana et al., 2019) and resistance exercises (Nygaard et al., 2015; Tsuchiya et al., 2015; Kim et al., 2016) lead to more significantly enhanced in irisin level, and improve bone loss in patients with osteoporosis (Watson et al., 2019). This means that irisin has become a new target in promoting bone health, and the possibility of treating some bone diseases.

Irisin is also considered a biomarker in the musculoskeletal system. In postmenopausal women, irisin can be used as a biomarker for sarcopenia and hip fracture, because irisin is inversely related to the degree of muscle wasting and the risk of hip fractures (Yan et al., 2018; Park et al., 2019; Ruan et al., 2020). In body composition of children, Irisin is also used as a biomarker owing to the positive correlation with BMD, regardless of lean or fat body mass (Eloranta et al., 2018). Remarkably, according to multiple regression analysis, irisin is even a stronger determinant of bone mineral status than bone alkaline phosphatase (Colaianni et al., 2019).

## Summary

**FIGURE 1:**
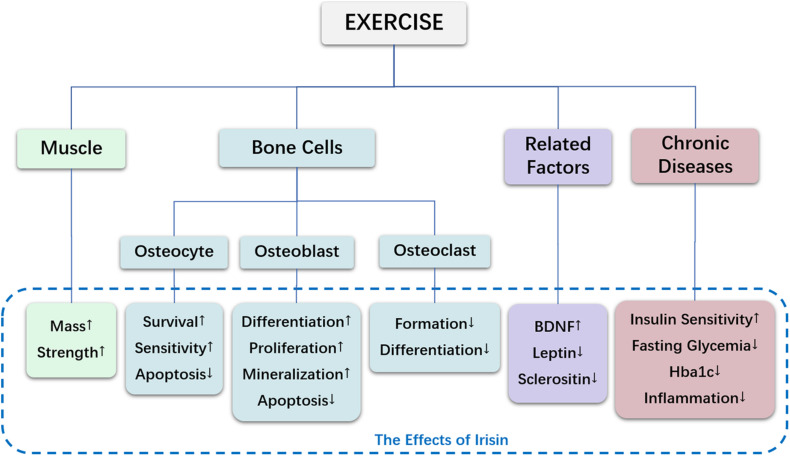
The effects of irisin in exercise-mediated bone metabolism.

Since the discovery of irisin, owing to many findings relevant to bone metabolism, irisin has attracted much attention. In exercise-mediated bone metabolism, irisin ameliorates bone metabolism by regulating muscle and bone cells, modulates the expression of cell factors, and alleviates bone loss under pathological conditions, as shown in Table 1 and Figure 1. Interesting, irisin is expected to serve as a biomarker for detecting bone metabolism. Finally, exercise is beneficial for maintaining bone health partly via regulation of irisin, and this may also apply to people with underlying pathological conditions.

**TABLE 1:** Irisin regulates bone metabolism by different factors.

Factor	Action	References
MAPK	Improve osteocyte survival, inhibit apoptosis	Storlino et al., 2019
P38 MAPK and ERK	Improve osteoblast osteogenic	Qiao et al., 2016
Nrf2	Inhibit osteoblast apoptosis	Xu et al., 2020
AMPK-α	Improve osteoblast differentiation	Ye et al., 2020
RANKL	Inhibit osteoclast differentiation	Zhang et al., 2017; Kawao et al., 2018
Sclerostin	Improve osteocyte survival and osteoblastogenesis	Colaianni et al., 2017; Kim et al., 2018
Muscle stem cell-derived factors	Increase muscle mass and strength	Reza et al., 2017b
PI3K	Improve insulin sensitivity, reduce fasting glycemia	Liu et al., 2015
NF-κB	Inhibit inflammation	Mazur-Bialy et al., 2017

## Author Contributions

JX and LL conceptualized the review. LL, JG, and XC wrote the manuscript. XT prepared the figure and table. JX and JZ critically reviewed and edited the manuscript. All authors contributed to the article and approved the submitted version.

## Conflict of Interest

The authors declare that the research was conducted in the absence of any commercial or financial relationships that could be construed as a potential conflict of interest.
